# FTO – A Common Genetic Basis for Obesity and Cancer

**DOI:** 10.3389/fgene.2020.559138

**Published:** 2020-11-16

**Authors:** Ning Lan, Ying Lu, Yigan Zhang, Shuangshuang Pu, Huaze Xi, Xin Nie, Jing Liu, Wenzhen Yuan

**Affiliations:** ^1^The First School of Clinical Medicine, Lanzhou University, Lanzhou, China; ^2^The First Hospital of Lanzhou University, Lanzhou, China; ^3^Key Laboratory for Resources Utilization Technology of Unconventional Water of Gansu Province, Gansu Membrane Science and Technology Research Institute Co., Ltd., Lanzhou, China; ^4^The Second Hospital of Lanzhou University, Lanzhou, China; ^5^Changjiang Scholar’s Laboratory/Guangdong Provincial Key Laboratory for Diagnosis and Treatment of Breast Cancer, Shantou University Medical College, Shantou, China

**Keywords:** obesity, cancer, FTO, SNP, M^6^A modification, mTOR, FTO inhibitors

## Abstract

In recent years, the prevalence of obesity and cancer have been rising. Since this poses a serious threat to human health, the relationship between the two has attracted much attention. This study examined whether fat mass and obesity-associated (*FTO*) genes are linked, taking into account a Genome-wide Association Study (GWAS) that revealed multiple single nucleotide polymorphism sites (SNPs) of the *FTO* gene, indicating an association between obesity and cancer in different populations. FTO proteins have been proved to participate in adipogenesis and tumorigenesis with post-transcriptional regulation of downstream molecular expression or through the target of the mammalian target protein rapamycin (mTOR). FTO inhibitors have also been found to share anti-obesity and anti-cancer effects *in vivo*. In this review, we comprehensively discuss the correlation between obesity and cancer by measuring *FTO* gene polymorphism, as well as the molecular mechanism involved in these diseases, emphasizing FTO as the common genetic basis of obesity and cancer.

## Introduction

The morbidity of obesity and cancer is increasing year by year in most countries around the world and represents a threat to human health (Ng et al., 2014; Siegel et al., 2018). Obesity causes changes in the body’s physiological and hormonal environments that promote many diseases, including diabetes and cardiovascular diseases. Obesity has been proven to increase the risks of at least 13 different types of cancers, such as esophageal adenocarcinoma, colon cancer, endometrial cancer, postmenopausal breast cancer, kidney cancer, and hematopoietic cancers (Calle and Kaaks, 2004; Goodwin and Stambolic, 2015). Of all Americans diagnosed with cancer in 2014, the overweight and obese population account for 40% (Steele et al., 2017). Furthermore, another prospective study of large samples of Americans confirmed that 14% of cancer deaths in males and 20% of females are due to them being overweight or having obesity (Calle et al., 2003). The biological mechanism of obesity and cancer are complex, including obesity-related hormones, growth factors, multiple signaling pathways, and chronic inflammation (Chen, 2011; Vucenik and Stains, 2012). In recent years, *FTO* SNPs have been firmly associated with increased body mass index (BMI) and higher risks of various types of cancers in people of multiple races, and the role of *FTO* SNPs in the development of obesity and cancer has been gradually revealed (Loos and Yeo, 2014; Hernández-Caballero and Sierra-Ramírez, 2015; Deng et al., 2018a; Chen and Du, 2019). This review details this role and the molecular mechanisms of *FTO* in obesity and cancer, as well as its potential clinical applications as a therapeutic target.

## *FTO* Gene and Functions

In 1999, *FTO* was first cloned by exon trapping analysis in Fused toes (Ft) mutation mice (Peters et al., 1999). Initially, *FTO* was expected to be associated with programmed cell death because scientists observed that heterozygous mice with Ft mutation developed syndactyly in the forelimb part and thymus hyperplasia (Van Der Hoeven et al., 1994). In 2007, the GWAS study identified *FTO* as an obesity sensitivity gene, and multiple SNPs in the intron 1 region were strongly associated with BMI, body fat rate, waist circumference, hip circumference, and energy intake (Dina et al., 2007; Frayling et al., 2007; Scuteri et al., 2007). As a result, the gene was named as the fat mass and obesity-associated (*FTO*) gene and has received extensive attention.

According to current genomics research, the *FTO* gene only exists in vertebrates and a few kinds of marine algae with highly conserved nucleotide and amino acid sequences (Robbens et al., 2008). The human *FTO* gene is located on chromosome 16q12.2, encoding a 2-oxoglutarate (2-OG) Fe(II)-dependent AlkB family dioxygenase, with a total length of 410.50 kb including 9 exons and 8 introns. About 3.4 kb upstream of *FTO* gene was Merkel’s diverticulum syndrome-associated gene (*RPGRIP1L*), and its downstream was close to Iroquois gene family (including *IRX3*, *IRX5*, *IRX6*) (Supplementary Figure). *FTO* is extensively expressed in adipose tissues and the skeletal muscles of human tissues, with the highest expression in the hypothalamus in the region that controls energy balance, namely the arcuate nucleus, which indicates that it may play a critical role in regulating appetite and energy metabolism (Frayling et al., 2007).

**FIGURE 1:**
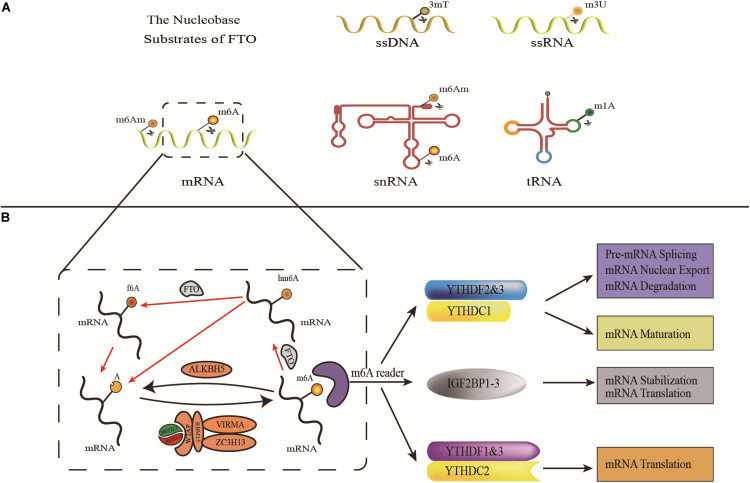
The demethylases activity of FTO. **(A)** The nucleobase substrates of FTO. **(B)** m^6^A modification is induced by METTL3, METTL14, and their cofactors(writers), reversed by FTO and ALKBH5 (erasers), and functionally facilitated by m^6^A binding proteins including YTHDF1-3, YTHDC1-2, and IGF2BP1-3(readers).

In 2007, Thomas et al. revealed that the *FTO* gene encodes Fe(II)/2-OG dependent demethylase, which is the ninth AlkB family protein found in mammals (also called ALKBH9) (Gerken et al., 2007). They also used purified FTO protein from recombinant mice or humans that can catalyze the demethylation of 3-methylthymine(3-meT) and 3-methyluracil (3-meU) with the help of Fe(II)/2-OG (Gerken et al., 2007; Jia et al., 2008). Later, He et al. found that N^6^-methyladenosine (m^6^A) in nuclear RNA was a main substrate of the FTO (Jia et al., 2011). Therefore, the FTO was identified as the first RNA demethylase, thus initiating a wave of research on epigenetic modifications of RNA. Since then, the complex and diverse functions of FTO proteins have been gradually revealed. FTO can bind to multiple types of RNAs, including mRNA, snRNA, and tRNA, and can demethylate m^6^A and N^6^,2′-O-dimethyladenosine (m^6^Am) in mRNA, m^6^A in U6RNA, m^6^Am in snRNAs, and N^1^-methyladenosine (m^1^A) in tRNA (Wei J. et al., 2018; Figure 1A). However, m^6^A is the most favorable nucleobase substrate of FTO (Zhang X. et al., 2019).

M^6^A, methylation modification on the sixth nitrogen atom of adenine (Wei et al., 1975) is the most common mRNA methylation enriched in the 3′-untranslated region (3′-UTRs), between the stop codon and the start codon (Roundtree et al., 2017). M^6^A modifications were subjected to reversible and dynamic regulations including writers (METTL3, METTL14, and WTAP), erasers (FTO and ALKBH5), and readers (YTH domain family and IGF2BPs) (Aik et al., 2014; Liu et al., 2014; Ping et al., 2014; Schwartz et al., 2014; Wang et al., 2014, 2015, 2016; Huang et al., 2018; Liao et al., 2018; Figure 1B). Based on this extensive existence and dynamic regulation, m^6^A plays an important role in post-transcriptional regulatory processes such as RNA splicing, nuclear production, degradation, and translation (Fustin et al., 2013; Wang et al., 2014, 2015; Bartosovic et al., 2017; Huang et al., 2018).

## Association of *FTO* SNPs With Obesity or Cancer

Since *FTO* has been identified as the first obesity-related gene. By conducting GWAS analysis, researchers have found that *FTO* SNPs are associated with obesity and higher risks of various cancers in multiracial populations (Supplementary Figure).

### Association of *FTO* SNPs With Obesity

The connection between *FTO* SNPs and BMI was first found in European people with diabetes. The classic BMI-related *FTO* SNPs were rs9939609 (T/A), and compared with those who did not carry the risk allele, 16% of adults who carried the homozygous risk allele gained nearly 3 kg in weight, and the risk of obesity increased by 1.67 times (Frayling et al., 2007). Serial GWAS studies on obesity-related traits in people of European descent have confirmed the important role of the *FTO* locus, and many other *FTO* SNPs in the intron 1 region have been reported, such as rs9930506, rs1421085, rs8050136, rs1121980, and so forth (Dina et al., 2007; Scuteri et al., 2007; Haupt et al., 2008; Cauchi et al., 2009). The obesity-associated *FTO* SNPs in East Asian populations are comparable to that of people of European descent. The risk allele A of *FTO* SNP rs9939609 was closely related to obesity and BMI in Chinese, ethnic Chinese, Malaysian, Singaporean, East, and South Asian people (Chang et al., 2008; Tan et al., 2008; Li et al., 2012). A large-scale meta-analysis targeting GWAS analysis of East Asian populations found that *FTO* SNPs rs17817449 have the most significant correlation with BMI in people of Chinese descent (Wen et al., 2012). In recent years, global studies have associated the rs9939609 variant with higher obesity risks in other populations (including Brazilian people, early adolescence in China, and adults in Shiraz, Iran) (Fonseca et al., 2019; Jiang et al., 2019; Mehrdad et al., 2020b). It is also associated with increased BMI and waist circumference (in Brazilian youths) (Reuter et al., 2016), adipose tissue distribution (in Italian people), and increased metabolic syndrome susceptibility (in Chinese populations) (Merra et al., 2020; Wang et al., 2020). In terms of the potential mechanisms between mutations in *FTO* and increased risks of obesity, studies have proven the role of *FTO* in the influence of food intake. People carrying *FTO* risk alleles and are inclined to higher energy intake foods like fat or proteins, reduced satiety, resulting in overeating, and many even lose control when eating (Cecil et al., 2008; Sonestedt et al., 2009; Tanofsky-Kraff et al., 2009; Ahmad et al., 2011). Another large-scale meta-analysis showed that the homozygous *FTO* risk allele was associated with a 27% lower risk of obesity in physically active adults (Kilpeläinen et al., 2011).

### Association of *FTO* SNPs With Cancer

To date, it has been studied that variants of *FTO* rs9939609, rs8050136, rs1477196, rs6499640, rs1121980, rs17817449, rs11075995, rs8047395, and rs7206790 have an association with a higher risk of cancers (Hernández-Caballero and Sierra-Ramírez, 2015). The most typical *FTO* SNP rs9939609 was associated with lung cancer, renal cancer, breast cancer, prostate cancer, pancreatic cancer, endometrial cancer (Delahanty et al., 2011; Kaklamani et al., 2011; Lin et al., 2013; Huang et al., 2017). Multiple SNPs in the intron 1 region of the *FTO* (including rs9939609, rs1477196, rs7206790, rs8047395) have been correlated with the risk of breast cancer, with rs1477196 strongly associated (Kaklamani et al., 2011). Interestingly, Da et al. observed that the interaction of *FTO* and *MC4R* polymorphisms showed a strong association with breast cancer: there was a 4.59-fold increased risks for women who have the allele combination C/T/C (*FTO* rs1121980/*FTO* rs9939609/*MC4R* rs17782313) (Da Cunha et al., 2013). In addition, in 2013, Iles et al. found an association between *FTO* rs16953002 and rs12596638 and melanoma susceptibility. However, these two SNPs are located in intron 8 of the *FTO* gene rather than intron 1 (the BMI-related region). This suggests that the association between the *FTO* variant and a wide range of diseases may play a role beyond BMI (Iles et al., 2013).

## FTO Is Involved in the Pathogenesis of Obesity and Cancer

### FTO, as the RNA m^6^A Demethylase, Is Involved in the Development of Obesity and Cancer

FTO proteins are widely involved in both adipogenesis and tumorigenesis by m^6^A-dependent demethylase activity which influences several mRNA processing events (Table 1).

**TABLE 1:** FTO proteins are widely involved in both adipogenesis and tumorigenesis by m^6^A-dependent demethylase activity.

**Disease**	**FTO biological function**	**Target RNA**	**References**
Obesity	FTO overexpression increased energy intake through reduce ghrelin mRNA m^6^A.	Ghrelin	Karra et al., 2013
Obesity	FTO regulates pre-adipocyte differentiation by regulating m^6^A levels around splice sites to control the splicing of the exon of adipogenic regulatory factor RUNX1T1.	SRSF2	Zhao et al., 2014
Obesity	FTO regulated adipogenesis by regulating cell cycle protein by m^6^A-YTHDF2 dependent pathway.	CCNA2, CDK2	Wu et al., 2018a,b
Acute myeloid leukemia (AML)	FTO enhanced leukemic cell transformation and leukemogenesis and limited all-*trans-*retinoic acid (ATRA)-induced AML cell differentiation.	ASB2, RARA	Li et al., 2017
Glioblastoma	FTO induced Glioblastoma Stem Cells (GSC)growth, self-renewal, tumor progression, and prolonged mouse lifespan by regulating m^6^A of cancer-associated genes.	ADAM19, EPHA3, KLF4, CDKN2A, BRCA2, TP53I11	Cui et al., 2017
Breast cancer	FTO promoted breast cancer cells malignant phenotypes such as proliferation, colony formation, and metastasis.	BNIP3	Niu et al., 2019
Gastric cancer (GC)	FTO knockdown increased m^6^A level promoting GC cell proliferation and invasion by activating Wnt and PI3K-Akt signaling.	Wnt/PI3K-Akt pathway	Zhang C. et al., 2019
Lung squamous cell carcinoma (LUSC)	FTO effectively promoted cell proliferation and invasiveness and inhibited cell apoptosis of lung squamous cells.	MZF1	Liu et al., 2018
Non-small cell lung cancer (NSCLC)	FTO promoted the proliferation, colony formation ability of lung cancer cells *in vitro*, and promoted lung cancer cell growth *in vivo*.	USP7	Li et al., 2019a
Hepatocellular carcinoma (HCC)	Knockdown of FTO suppressed the proliferation and *in vivo* tumor growth, and induced the G0/G1 phase arrest.	PKM2	Li et al., 2019b
Cervical squamous cell carcinoma (CSCC)	FTO increased β-catenin mRNA expression, increased DNA repair activity, and induced resistance to chemoradiotherapy.	β-catenin	Zhou et al., 2018
Leukemia	The demethylation mediated by FTO promoted the stability of proliferation-related genes.	MERTK, BCL-2	Yan et al., 2018
Melanoma	FTO accelerated melanoma tumorigenesis and anti-PD-1 resistance by regulating the expression of critical cell-intrinsic genes in an m^6^A-YTHDF2 dependent manner.	PD-1 (PDCD1), CXCR4, SOX10	Yang et al., 2019

#### FTO, as the RNA m^6^A Demethylase, Is Involved in the Development of Obesity

**FIGURE 2:**
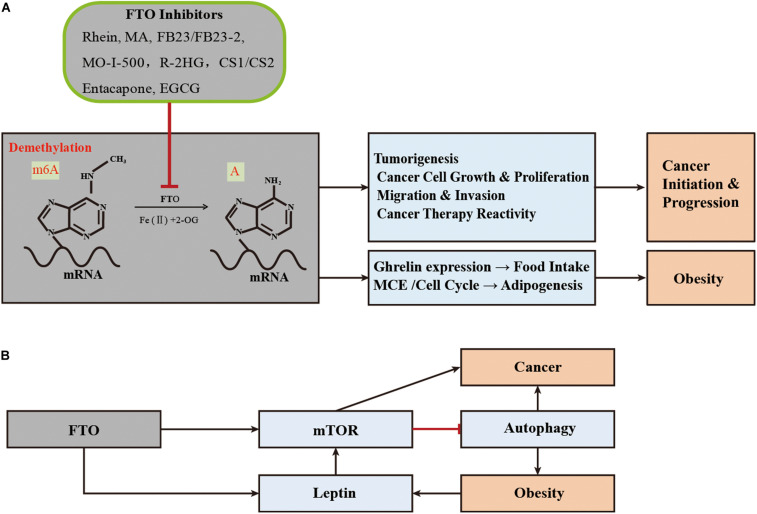
Schematic illustration of the roles of FTO in obesity and tumorigenesis/drug response. **(A)** As the m^6^A demethylase, FTO post-transcriptionally regulates expression of its critical target genes and thereby contributes to obesity (through affecting adipogenesis and food intake) and cancers (including tumorigenesis, cancer cell growth, migration, and drug response). MA, meclofenamic acid; R-2HG, 2-hydroxyglutarate; EGCG, Epigallocatechin gallate; **(B)** FTO regulates the development and progression of obesity and cancer through the mTOR or adipofactor-mTOR pathway.

FTO proteins are involved in the development of obesity by affecting the m^6^A level of hormones related to eating or molecules related to adipogenesis (Figure 2A). In 2013, Efthimia et al. found that FTO over-expression limited the m^6^A modification of ghrelin mRNA in cell models and increased ghrelin mRNA and peptide levels concomitantly. This article provided insights into how FTO predisposes to stimulated energy intake and obesity in humans (Karra et al., 2013). Simultaneously, substantial evidence has proved that FTO participates in the process of adipogenesis (Ben-Haim et al., 2015). Zhao et al. found that FTO regulates the exonic splicing of the adipogenic regulator RUNX1T1 by influencing the level of m^6^A around the splice site, thereby modulating cell differentiation (Zhao et al., 2014). Moreover, FTO affects adipogenesis by regulating the process of mitotic clonal expansion (MCE), which is a prerequisite for adipocyte differentiation that occurs within 48 h of adipogenic stimulation (Merkestein et al., 2015). The overexpression of FTO can induce MCE and regulate the differentiation of preadipocytes by influencing the expression of m^6^A-dependent transcription factors (Tang et al., 2003; Merkestein et al., 2015; Zhang et al., 2015). Furthermore, Wu et al. found that FTO regulated adipogenesis by dominating cell cycle proteins such as CCNA2 and CDK2 by m^6^A-YTHDF2 dependent pathway, revealing a new mechanism about anti-obesity and anti-adipogenesis activity of Epigallocatechin gallate (EGCG) (Wu et al., 2018a,b).

#### FTO, as the RNA m^6^A Demethylase, Regulates the Malignant Phenotype and Therapeutic Response of Cancer Cells

FTO is highly expressed in many cancer tissues, which plays the role of an oncogene in an m^6^A-dependent way and participates in the regulation of the malignant phenotype of cancer cells (Figure 2A). In 2017, Li et al. found that FTO enhanced leukemia oncogene-mediated cell transformation and leukemogenesis and inhibited all-*trans-*retinoic acid (ATRA)-induced AML cell differentiation by affecting the expression of targets mRNAs such as ASB2 and RARA, through reducing the level of m^6^A (Li et al., 2017). Moreover, the elevated level of m^6^A was found to promote the growth, self-renewal, and tumorigenesis of Glioblastoma Stem Cells (GSC) as well as prolong the lifespan of GSC-grafted mice (Cui et al., 2017). In 2018, Niu et al. showed that FTO can promote breast cancer cell malignant phenotype through epigenetically demethylated m^6^A in BNIP3 mRNA 3′UTR (Niu et al., 2019). Similarly, the overexpression level of FTO was also found in gastric cancer, advanced non-small cell lung cancer, and hepatocellular carcinoma, which can regulate cell proliferation and/or migration/invasion through targeting demethylation for the m^6^A of Wnt/PI3K-Akt, USP7, MZF1, or PKM2, respectively (Liu et al., 2018; Li et al., 2019a,b; Zhang C. et al., 2019).

FTO may also have an impact on the therapeutic response of cancer (Figure 2A). In 2018, Zhou et al. found that FTO enhanced the chemo-radiotherapy resistance of cervical squamous cell carcinoma both *in vitro* and *in vivo* through influencing the expression of ß-catenin by reducing m^6^A levels (Zhou et al., 2018). The overexpression of FTO in leukemia cells can promote its expression by catalyzing the demethylation of cell proliferation-related genes such as m^6^A of MERTL and BCL-2 mRNA and affecting the generation of resistance phenotypes in the treatment with tyrosine kinase inhibitors (TKI) (Yan et al., 2018). Recently, He et al. found that the down-regulation of FTO made melanoma cells sensitive to interferon-gamma (IFN-γ) and anti-PD-1. FTO played a crucial role by promoting melanoma tumorigenesis and anti-PD-1 resistance (Yang et al., 2019).

### FTO-mTOR Axis Affects Obesity and Cancer

The mammalian target protein rapamycin (mTOR) is an atypical serine/threonine kinase, which is the core component of regulating mRNA translation and can promote cell growth according to environmental signals (Laplante and David, 2012). mTOR binds to a variety of chaperone proteins to form two different kinase complexes, i.e., mTOR complex 1 (mTORC1) and mTORC2.

In 2013, *in vivo* experiments demonstrated that FTO played a role in the coupling of amino acid level and mTORC1 signaling pathway. FTO deficient cells showed reduced activation of the mTORC1 pathway, decreased mRNA translation rate, and increased autophagy (Cheung et al., 2013; Gulati et al., 2013). mTORC1 is a negative regulator of autophagy, which is a major cellular digestion process. In response to nutrition and environmental stress, autophagy plays a critical role in the occurrence and progression of obesity and cancer (White and Dipaola, 2009; Kim and Guan, 2015; Zhang Y. et al., 2018). Furthermore, mTOR activates the Warburg effect by inducing PKM2 and other glycolytic enzymes under normoxic conditions (Sun et al., 2011). In summary, mTORC1 can regulate the development of obesity and cancer through autophagy or direct activation of downstream signaling pathways (Laplante and David, 2012).

Mutations in the *FTO* gene raise blood levels of leptin, a known mediator or growth factor between obesity and colon cancer, which activates a variety of pathways associated with colon cancer (Drew, 2012; Mehrdad et al., 2020a). In addition, leptin has been suggested as an intermediate link between obesity and breast or prostate cancer (Stattin et al., 2001; Barone et al., 2020). Intriguingly, mTOR is also one of the signal mediators of obesity related factors, such as leptin, adiponectin, and inflammatory cytokines, through the Akt/PI3K or AMPK pathways (Maya-Monteiro and Bozza, 2008; Wang et al., 2012; Mauro et al., 2018). This seems to coincide with the FTO-mTOR pathway, discussed in the previous paragraph. In summary, FTO can directly or indirectly target mTOR, thus regulating the occurrence and progress of obesity and cancer in a broad manner (Figure 2B).

## Effects of FTO Inhibitors in Obesity and Cancer

With the gradual disclosure of the important functions of FTO as mRNA demethylase in many diseases, the crystal structure of FTO has been resolved since 2010, and the development and applications of its specific inhibitors have attracted extensive attention (Han et al., 2010). FTO inhibitors that have been shown to have anti-obesity or anti-cancer effects *in vitro* or *in vivo* are summarized in Table 2.

**TABLE 2:** Summary of the effects and application of FTO inhibitors in obesity and cancer.

**Inhibitor**	**Mechanisms for inhibiting FTO**	**Specific inhibition?**	**The mechanisms of anti-cancer effect**	**Anti-obesity?**	**References**
Rhein	Rhein reversibly binds to the FTO enzyme, competitively preventing the recognition of the m^6^A substrate.	No	Rhein can be used in combination with nilotinib to inhibit the progression of leukemia in mice; Rhein inhibited subcutaneous breast tumor growth in mice.	Rhein has anti-obesity effect, but it needs to be further clarified whether by inhibiting FTO.	Liu et al., 2011; Chen et al., 2012; Zhang et al., 2012; Yan et al., 2018; Niu et al., 2019
MA/MA2	MA/MA2 competed with FTO to bind with m^6^A.	Yes	MA2 inhibited the progression of glioblastoma and extended the life span of GSC transplanted animals.	Unknown	Huang et al., 2015; Cui et al., 2017
FB23/FB23-2	FB23/FB23-2 binds to FTO and selectively inhibits the m^6^A demethylase activity of FTO.	Yes	FB23 and FB23-2 significantly increased the abundance of ASB2 and RARA and inhibited MYC and CEBPA expression in AML cells.	Unknown	Huang et al., 2019
R-2HG	R-2HG is structurally close to 2-OG so that it can competitively inhibit FTO.	No	R-2HG can inhibit leukemia and glioma through the regulation of R-2HG-FTO-m^6^A axis to MYC/CEBPA expression and downstream pathways.	Unknown	Su et al., 2018
CS1/CS2	Direct interaction between CS1/CS2 and intracellular FTO protein inhibits its demethylase activity.	Unknown	CS1 and CS2 play an anti-leukemic role by manipulating FTO-related signaling pathways, such as the MYC pathway.	Unknown	Su et al., 2019
MO-I-500	MO-I-500 is a mimic of 2-OG, which can inhibit the RNA demethylase activity of FTO and increase the content of m^6^A in the total RNA of cells.	Yes	As a pharmacological inhibitor of FTO, MO-I-500 plays an important role in the cell survival of refractory triple-negative inflammatory breast cancer.	Unknown	Zheng et al., 2014; Singh et al., 2016
Epigallocatechin gallate (EGCG)	EGCG will reduce the protein stability of FTO and affect its protein expression.	No	EGCG has an anti-cancer effect, but it needs to be further clarified if it through inhibiting FTO.	EGCG prevents mitotic cloning amplification (MCE) at the early stage of adipocyte differentiation by inhibiting FTO expression.	Stuart et al., 2006; Forester and Lambert, 2014; Negri et al., 2018; Wei R. et al., 2018; Wu et al., 2018b; La et al., 2019; Wei et al., 2019; Zhang L. et al., 2019
Entacapone	Entacapone can directly combine with FTO and inhibit the activity of FTO.	Yes	Entacapone has an anti-cancer effect, but it needs to be further clarified if it through inhibiting FTO.	Entacapone has an effect on gluconeogenesis and adipose tissue heat production in mouse liver by acting on the FTO-FOXO1 axis.	Forester and Lambert, 2014; Peng et al., 2019

### Effects of FTO Inhibitors in Obesity

In 2012, Yang et al. reported natural product Rhein as an inhibitor of human FTO demethylase, which can competitively bind to the FTO catalytic site (Chen et al., 2012). Before this study, Rhein was thought to prevent or even reverse weight gain and obesity caused by high-fat diets (Liu et al., 2011; Zhang et al., 2012). The catechin EGCG, another natural compound rich in green tea, was found to play anti-obesity and anti-adipogenesis roles through the FTO-m^6^A-YTHDF2 axis (Wu et al., 2018b). Recently, Peng et al. identified Entacapone as a potential FTO inhibitor, which has the effect of reducing weight and lowering blood glucose (Peng et al., 2019). It was initially approved as an adjunctive therapy combined with levodopa and carbidopa for the treatment of Parkinson’s disease. Entacapone had an effect on gluconeogenesis and adipogenesis in the liver of mice by acting on an FTO-FOXO1 regulatory axis (Peng et al., 2019).

### Effects of FTO Inhibitors in Cancer

As for cancers, there are more studies on the application of FTO inhibitors, especially in the treatment of leukemia, glioblastoma, and breast cancer. Compared with single drug therapy, Rhein combined with nilotinib is a more effective treatment for leukemia in mice (Yan et al., 2018). Recently, Yang et al. identified meclofenamic acid (MA) as a highly selective inhibitor of FTO, which can compete with FTO binding for the m^6^A-containing nucleic acids (Huang et al., 2015). The inhibitor FB23 was designed and synthesized from the chemical scaffold of MA, which exhibited a more potent inhibition for FTO demethylation *in vitro* (Huang et al., 2019). Its bioisostere FB23-2 can inhibit the leukemogenesis in cells and in the patient-derived xenografted (PDX) mouse model (Huang et al., 2019). Su et al. found that R-2HG (oncometabolite produced by mutant isocitrate dehydrogenase 1/2 (IDH1/2) enzymes), compounds CS1 and CS2 were also targeted inhibitors of FTO. By inhibiting its demethylation function, they affected related signaling pathways (such as the MYC pathway) and played an active role in inhibiting the proliferation of AML cells *in vivo* and *in vitro* (Su et al., 2018, 2019). Compounds CS1 and CS2 extended the overall survival of transplanted mice with primary MLL-AF16 cells and made AML cells sensitive to other curative drugs, such as decitabine, a tyrosine kinase inhibitor, and IDH2^*mut*^ inhibitor (Su et al., 2019). Comparing the anti-leukemic activities of the four FTO inhibitors, CS1 and CS2 showed higher activity in inhibiting cell viability, and their IC50 values were 10–30 times lower than FB23-2 or MO-I-500 (Su et al., 2019). MO-I-500 is another FTO inhibitor, which selectively inhibits the demethylation of FTO and increases the m^6^A levels in cells (Zheng et al., 2014). In addition, among the above-mentioned FTO inhibitors, Rhein and MO-I-500 have been reported to significantly inhibit the growth ability of breast cancer cells *in vivo* and *in vitro* (Singh et al., 2016; Niu et al., 2019). MA2 (the ethyl ester form of MA) and R-2HG had inhibitory effects on glioma (Cui et al., 2017; Su et al., 2018; Deng et al., 2018b). Compared with MA, MA2 has a better cell penetration, significantly increased m^6^A methylation in cells, suppresses glioblastoma progression, and prolongs the lifespan of GSC-grafted animals (Huang et al., 2015; Cui et al., 2017). Interestingly, the previous anti-obesity EGCG, Entacapone, also had an inhibitory effect on cancer. For example, EGCG had an inhibitory effect on lung cancer, breast cancer, colon cancer, metastatic pancreatic cancer, and prostate cancer or had a sensitivity to chemotherapy. It was noted that it can be used as an adjuvant drug in cancer treatments (Negri et al., 2018; Wei R. et al., 2018; La et al., 2019; Wei et al., 2019). The combination of Entacapone and EGCG can synergistically enhance the growth inhibitor of lung cancer cell lines (Forester and Lambert, 2014).

In summary, more FTO inhibitors are displaying positive therapeutic effects in animal disease models, and represent promising therapeutic targets for obesity and cancer (Figure 2A).

## Controversy on the Mechanism of Association Between *FTO* Risk Alleles and Diseases

Single nucleotide polymorphism sites are the main form of human genome DNA sequence variation and can regulate gene expression. From *FTO* polymorphisms which have a risk for obesity and cancer to the specific mechanisms regulating these diseases through nucleic acid demethylation of FTO proteins, *FTO* SNPs seem to regulate the expression level of FTO and affect its enzymatic function, playing an important role in obesity and cancer. Some studies support this hypothesis. For example, In 2008, a study of a Mexican population revealed that in obese patients, the *FTO* risk allele was significantly correlated with high FTO expression (Villalobos-Comparán et al., 2008). Subsequently, Tea et al. and Efthimia et al. revealed that *FTO* mRNA caused by the risk allele was more abundant than non-risk alleles at least in blood cells (Berulava and Horsthemke, 2010; Karra et al., 2013). Unfortunately, the mechanism for the correlation between *FTO* SNPs and obesity or cancer has been elusive. So far, there are no studies that provide indisputable evidence for these associations.

**FIGURE 3:**
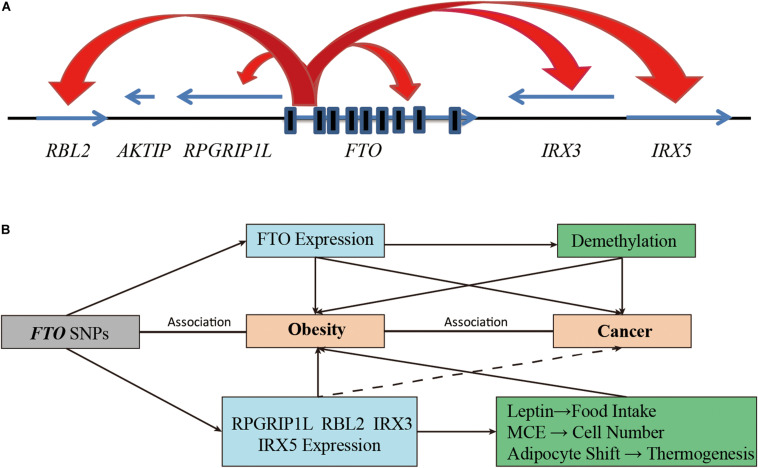
Increasing functional complexity around the FTO Locus. **(A)** SNPs in the intron 1 of *FTO* can regulate the expression of FTO itself and adjacent genes (including *RBL2, RPGRIP1L, IRX3*, and *IRX5*). **(B)** The *FTO* SNPs are involved in the development of obesity and cancer by affecting the demethylation function of FTO or by regulating the expression of adjacent genes.

Moreover, some studies have suggested that *FTO* SNPs may be associated with obesity by regulating the expression of adjacent genes (Tung et al., 2014; Figure 3A). The Leibel group found that the rs8050136 of the intron 1 region of *FTO* overlapped with the binding site of transcription factor Cut Like Homeobox 1 (CUX1). This SNP nucleotide type can affect the transcriptional activation of *FTO* and retinitis pigmentosa GTPase regulator interacting protein 1 like (*RPGRIP1L*) by CUX1 P110 (Stratigopoulos et al., 2008, 2011). For individuals with the obesity risk allele at rs8050136, the expression of *RPGRIP1L* and *FTO* in the hypothalamus were decreased due to the low binding affinity of CUX1 P110 to DNA, RPGRIP1L can affect the location of leptin receptors and leptin signaling in neurons and lead to increased food intake and obesity (Stratigopoulos et al., 2011). In addition, Jowett et al. found that in combination with gene variation and expression data from the human cohort, the A allele of rs8050136 was positively associated with the expression level of *RBL2*, and an increase in RBL2 level might help to restrict the clonal expansion of A population of precursor adipose cells during development (Jowett et al., 2010). Moreover, Smemo et al. found that these sites also contained an enhancer sequence that can bind to the promoter of *IRX3*. Using expression quantitative trait loci (eQTL), they found that obesity-related SNPs such as rs9930506 were correlated with the expression of *IRX3* in human brain samples. Mice lacking *IRX3* lost 25–30% of their body weight through increasing basal metabolic rate and browning of white adipose tissues (Smemo et al., 2014). The study of Claussnitzer et al. also supported the regulatory relationships between *FTO* SNPs and *IRX3* expression. They suggested that changes in the rs1421085 risk allele led to a double expression of *IRX3* and *IRX5* through disruption of the conservative motif of the ARID5B repressor in the early stage of adipocyte differentiation. In this case, brown fat cells transform automatically into white fat cells and lower down the mitochondrial thermogenesis by five times (Claussnitzer et al., 2015). This study explained the correlation between *FTO* SNPs and obesity by using the effect of the autonomous transformation of fat cells on thermogenesis. These studies provided a plausible mechanism for the correlation between SNP variation of *FTO* intron 1 and obesity (Figure 3B).

Adjacent genes (*RPGRIP1L, RBL2, IRX3, IRX5*) regulated by *FTO* SNPs are also involved in the occurrence and progression of cancer in various ways (Figure 3B). For example, studies have shown that RPGRIP1L was one of the tumor suppressor genes of human hepatocellular carcinoma (Lin et al., 2009). RBL2, a member of the retinoblastoma (Rb) family, was inactivated by cell cycle kinases and was the basis of various cancer types (Pentimalli et al., 2015). The emerging role of RBL2 in aging and apoptosis also appeared to play an active part in tumor inhibition (Pentimalli et al., 2015). IRX3 was highly expressed in approximately 30% of patients with AML, and high expression of IRX3 alone can perpetuate hematopoietic stem cells and progenitor cells (HSPC) in bone marrow cultures and induce lymphoid leukemia *in vivo* (Somerville et al., 2018). In addition, IRX3 and IRX5 have been reported to participate in the transition from intestinal adenoma to colorectal cancer by negatively regulating the Dpp/TGF-ß pathway (Martorell et al., 2014). IRX5 alone has also been reported to be an oncogenic gene in hepatocellular carcinoma, colorectal cancer, prostate cancer, and non-small cell lung cancer by regulating cancer cell cycle and apoptosis (Myrthue et al., 2008; Zhang D.L. et al., 2018; Zhu et al., 2019, 2020). Although there is no direct evidence that *FTO* SNPs are associated with cancer by affecting the expression of adjacent genes, we cannot rule it out as a possibility.

Based on these findings, the correlation between *FTO* SNPs and obesity and cancer may be due to the regulation of FTO enzyme activity or expression of adjacent genes. However, more convincing and systematic research studies are needed to decipher the causal mechanism between *FTO* non-coding variants and obesity or cancer. A healthy lifestyle such as proper diet and moderate exercise is recommended to minimize the negative effects of obesity susceptibility genes before we can fully understand the underlying mechanisms.

## Conclusion

Although the specific mechanisms for *FTO* polymorphism and high risk of obesity and cancer are elusive, the correlation is definite. As for FTO, it can participate in the disease progression of obesity and cancer in m^6^A-dependent post-transcriptional regulation, or by targeting mTOR. More importantly, some drugs have been shown to inhibit obesity, and some cancers such as leukemia, glioblastoma, and breast cancer by targeting FTO. In particular, FB23, R-2HG, and CS1/CS2 have shown anti-leukemia effects through *in vivo* experiments, and MA2 can also inhibit the activities of glioblastoma cells *in vivo*. This evidence suggests *FTO* as the common genetic basis of obesity and cancer and a potential target for obesity and some cancers.

## Author Contributions

WY oversaw and guided the process of writing this manuscript. NL wrote and edited the manuscript. YL, YZ, SP, XN, HX, and JL put forward suggestions for the manuscript. All authors read and approved the final manuscript.

## Conflict of Interest

The authors declare that the research was conducted in the absence of any commercial or financial relationships that could be construed as a potential conflict of interest.
