# Supplementary material for: FTO – A Common Genetic Basis for Obesity and Cancer
Source: Front Genet. 2020 Nov 16;11:559138. doi: 10.3389/fgene.2020.559138 (PMC7701174; doi:10.3389/fgene.2020.559138)
Supplement: Supplementary file 1 [file Table_1.DOCX]

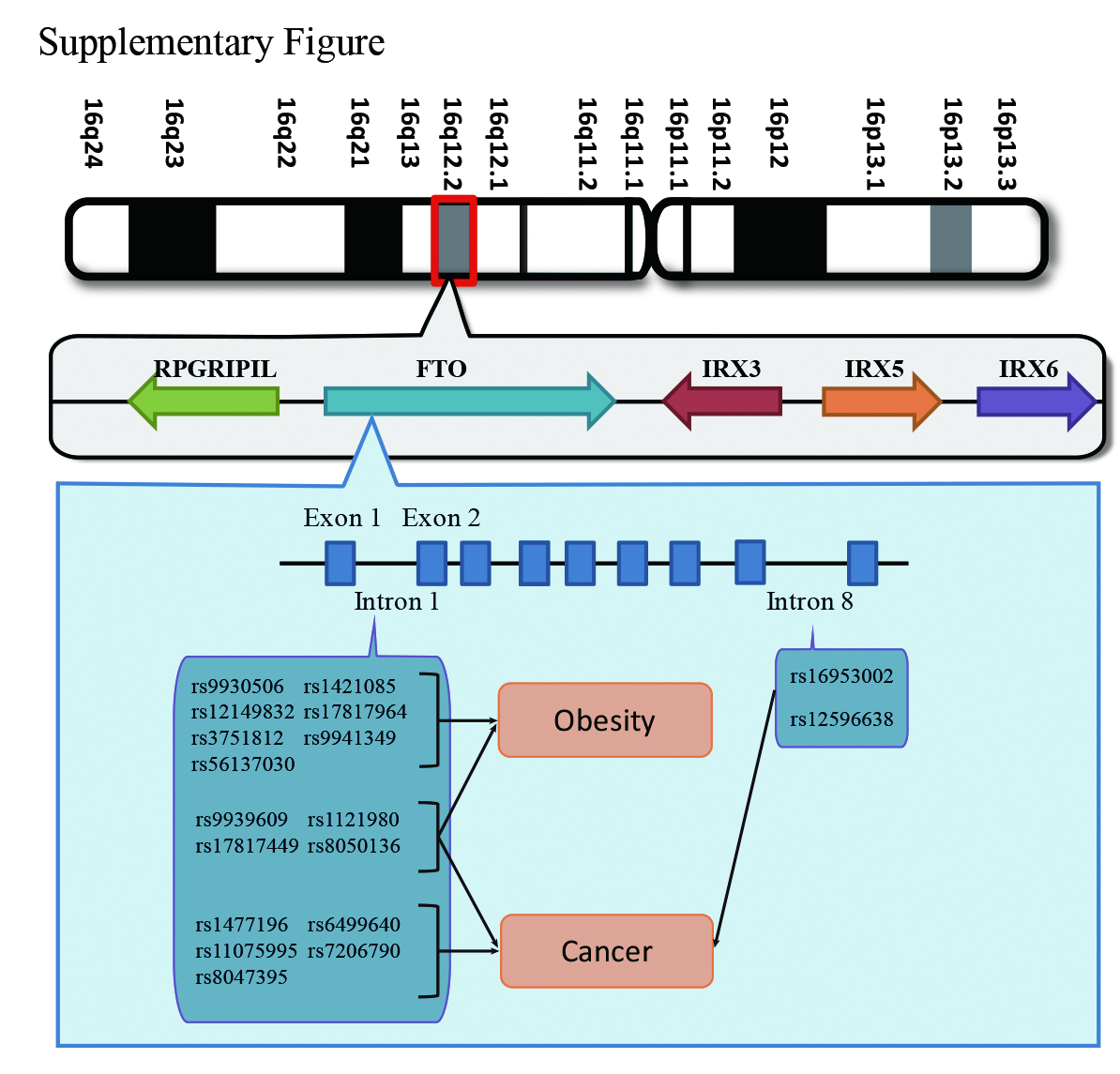


**Supplementary Figure.** Schematic diagram of *FTO* and its neighboring genes’ chromosomal location. SNPs in intron 1 or intron 8 which have a strong association with obesity or cancer were listed in below box.
